# Cardiovascular risk at health checks performed opportunistically or following an invitation letter. Cohort study

**DOI:** 10.1093/pubmed/fdx068

**Published:** 2017-06-17

**Authors:** Martin C Gulliford, Bernadette Khoshaba, Lisa McDermott, Victoria Cornelius, Mark Ashworth, Frances Fuller, Jane Miller, Hiten Dodhia, Alison J Wright

**Affiliations:** 1 Department of Primary Care and Public Health Sciences, King's College, London, UK; 2 NIHR Biomedical Research Centre at Guy's and St Thomas’ Hospital, London, UK; 3 Public Health Directorate, Lewisham Borough Council, London, UK; 4 Public Health Directorate, Lambeth Borough Council, London, UK

**Keywords:** cardiovascular disease prevention, health check, mass screening methods, primary care, social inequalities

## Abstract

**Background:**

A population-based programme of health checks has been established in England. Participants receive postal invitations through a population-based call–recall system but health check providers may also offer health checks opportunistically. We compared cardiovascular risk scores for ‘invited’ and ‘opportunistic’ health checks.

**Methods:**

Cohort study of all health checks completed at 18 general practices from July 2013 to June 2015. For each general practice, cardiovascular (CVD) risk scores were compared by source of check and pooled using meta-analysis. Effect estimates were compared by gender, age-group, ethnicity and fifths of deprivation.

**Results:**

There were 6184 health checks recorded (2280 invited and 3904 opportunistic) with CVD risk scores recorded for 5359 (87%) participants. There were 17.0% of invited checks and 22.2% of opportunistic health checks with CVD risk score ≥10%; a relative increment of 28% (95% confidence interval: 14–44%, *P* < 0.001). In the most deprived quintile, 15.3% of invited checks and 22.4% of opportunistic checks were associated with elevated CVD risk (adjusted odds ratio: 1.94, 1.37–2.74, *P*  < 0.001).

**Conclusions:**

Respondents at health checks performed opportunistically are at higher risk of cardiovascular disease than those participating in response to a standard invitation letter, potentially reducing the effect of uptake inequalities.

## Introduction

A programme of health checks for cardiovascular risk assessment has been established in England since 2011.^1^ The programme has proved controversial because of the limited evidence for the potential effectiveness of health checks^2,3^ and the arguably low cost-effectiveness when compared with population-wide intervention strategies.^4^ A programme of health checks might also exacerbate inequalities in cardiovascular disease if uptake of health checks is lower in groups at greater risk of cardiovascular disease.^5^

Implementation of the national health check programme is tailored flexibly in local areas. The health check programme in South London employs a population-based call–recall system, based on general practice population registers. Standard invitation letters are sent to eligible participants who are offered the choice of attending for a health check at their general practice or at selected local pharmacies and community settings.^1^ During the study period up to 25% of checks were performed by these ‘third party’ providers.^6^ Health check providers may also offer health checks during face-to-face encounters with patients who are attending for other reasons. These ‘opportunistic’ health checks contribute to the overall assessment of health check uptake.

The use of opportunistic health checks might have potential either to reduce or exacerbate inequalities in health check uptake. It is not known whether health checks conducted opportunistically differ, with respect to cardiovascular risk outcomes, when compared with health checks performed following standard invitation letters sent through the population-based call–recall system. This research aimed to compare cardiovascular risk estimates for health checks conducted either opportunistically or through the population-based invitation system.

## Methods

A cohort study was conducted of all health checks conducted between July 2013 and June 2015. The study was conducted alongside a randomized trial of enhanced invitations methods; the protocol and results of the trial have been reported previously.^6,7^ The research was approved by the London Bridge Research Ethics Committee on seventh March 2013 (reference13/LO/0197). All 18 general practices that participated in the trial were included in the analysis. These were selected from a wider pool of 89 general practices in the two boroughs. Trial practices tended to have larger list sizes than non-trial practices but achievement of Quality and Outcome Framework targets was generally similar between trial and non-trial practices. Trial practices showed similar levels of deprivation and a similar proportion of non-white participants to the entire populations of the two boroughs.^6^ Stratified randomization was employed to ensure that equal proportions were allocated to trial arms at each general practice. The study was conducted in two London Boroughs, which are generally deprived areas of inner London with young, ethnically diverse populations.^8^ All general practices in the two participating Boroughs were eligible and 18 consented to participate in the study. Each practice participated in the study for a minimum of 12 months to allow for seasonal variation in uptake of health checks. We evaluated health checks completed in participants invited up to 31st December 2014, in order to allow assessment of health check uptake at 6 months following the invitation up to 30th June 2015.

Records of health check uptake were extracted from participant electronic health records by members of the research team using nationally specified Read medical terms. At the time of data extraction, participants’ postcodes were linked to the Indices of Multiple Deprivation 2010 score (IMD 2010) as a marker of deprivation.^9^ Data for gender, year of birth and practice-recorded ethnicity were also extracted. We also extracted CVD risk score and body mass index records for registered patients who had a health check recorded during the study period. We obtained records of CVD risk scores that were coded into the electronic records of general practice systems. We did not extract data for individual CVD risk factors, including blood pressure, total cholesterol and smoking because our governance approvals did not extend to this. At the time of the study, the Joint British Societies^10^ (‘JBS3’) risk score calculator was mandated by the NHS Health Check Programme locally. Values for QRisk2^11^ score were utilized if the JBS3 score was not recorded. The JBS3 risk calculator is a CVD risk score developed for use in the UK that is based on smoking status, systolic blood pressure, total and HDL cholesterol and body mass index.^10^ The JBS3 score may generally give slightly lower risk estimates than QRisk2.^12^ We excluded duplicate records and analysed the first recorded risk score for each participant.

Health checks were classified into ‘invited’, those performed in participants who were sent a standard invitation letter during the study period, regardless of time interval; and ‘opportunistic’, those performed in participants who were not sent a standard invitation. The relative contribution of invited and opportunistic health checks to overall health check uptake was estimated by general practice. In order to estimate the pooled relative rate, while presenting data for each practice separately, a meta-analysis was conducted and a Forest plot was constructed. We compared the case mix of invited and opportunistic checks in terms of age-group, gender, ethnic group and deprivation quintile. We also compared CVD risk score estimates and body mass index category between invited and opportunistic checks. CVD risk was divided into the categories <10% or ≥10% risk of a CVD event over 10 years. Dichotomization at a clinically relevant cut-point was employed because the distribution of CVD risk scores is generally highly skewed. A cut-point of 20% was also used but only 6% of health checks were associated with ≥20% risk. Multiple logistic regression was employed to estimate adjusted odds ratios because binary regressions to estimate adjusted rate ratios did not converge. We used robust variance estimates to allow for correlation of measures by general practice.

Sensitivity analyses were conducted to explore the effect of varying the definition of ‘invited health’ checks and to evaluate the possible implications of health checks that were recorded without documentation of cardiovascular (CVD) risk scores. ‘Opportunistic health checks’ included 1 363/3 113 (44%) completed within 6 months of the practice start date in the study, these might potentially have resulted from invitations sent before the practice entered the study. The effect of excluding these was considered. To evaluate the effect of missing CVD risk score data, we divided general practices into groups with either lower (<85%) or higher (≥85%) recording of CVD risk scores at opportunistic health checks. We compared estimated associations for the two groups of practices.

The reliability of study data were evaluated by comparing results extracted directly from general practice records with data obtained from the local health check management information system. During the period up to 31st December 2015, the health check management information system recorded 12 453 participants invited, with 49% of completed health checks being performed opportunistically.

## Results

During the study period, 12 643 participants were invited through the population-based call–recall system, 2280 of these participants had health checks recorded. A total of 6184 health checks were recorded at study practices during the study, including 3904 in participants who were not sent a standard invitation letter. Cardiovascular risk score records were obtained for 5359/6184 (87%) health checks, including 2246/2280 (99%) of health checks in invited participants and 3113/3904 (80%) of health checks in non-invited participants. The 3113/5359 (58%) cardiovascular risk assessments in non-invited participants were either performed opportunistically, or were in patients invited before their general practice joined the study. These will be referred to as ‘opportunistic health checks’. There were 758 (14%) of records with missing JBS3 risk scores and QRisk2 scores were used for 524 (10%), leaving 234 (4%) with unclassified values that were included in the denominator.

We evaluated the distribution of invited and opportunistic health checks according to gender, age-group, ethnicity and deprivation quintile. The proportion of opportunistic checks was similar in men and women and across different ethnic groups. Opportunistic checks were more frequent in participants aged <60 years (2 703/4 583, 59%) than in those aged 60 years and older (410/776, 53%) with invited checks being more frequent in the older age group. In the study area, 85% of all participants were in the two lowest fifths of deprivation for England. Opportunistic checks were more frequent in the most deprived fifth (1028/ 1723, 60%) than in the third (290/525, 55%) or second least deprived fifth (2/17, 12%). However, evaluation of a possible linear trend showed an odds ratio of 0.92 (0.82–1.03, *P* = 0.137) for unit decrease in deprivation fifth from most to least deprived.

**Fig. 1:**
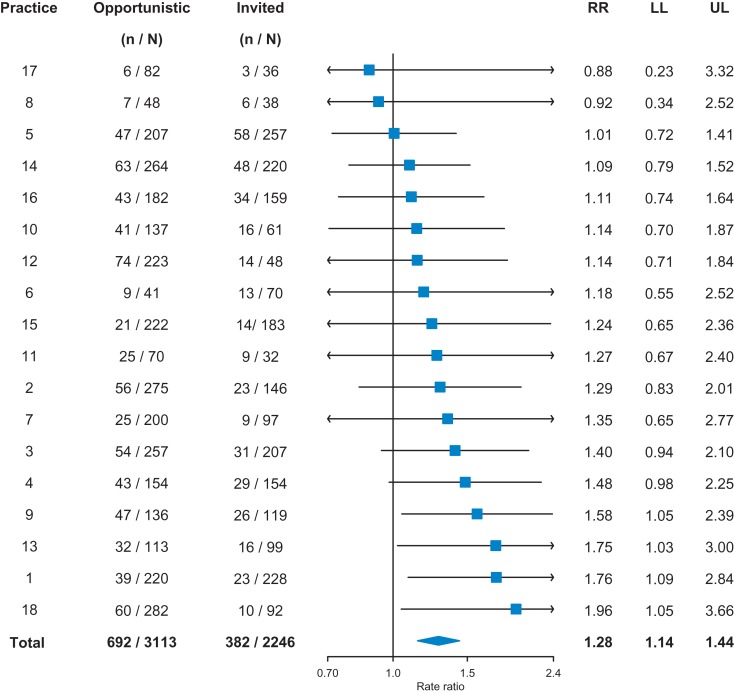
Forest plot showing the proportion with CVD risk ≥10% at each practice by source of health check. *N*, total number of checks; *n*, number with ≥10% CVD risk; RR, rate ratio; LL, lower 95% confidence limit; UL, upper 95% confidence limit.

Figure 1 presents the proportion of checks with CVD risk score of 10% or greater by general practice and source of health check. Overall, 382/2246 (17.0%) of invited checks and 692 (3113) 22.2% of opportunistic health checks were associated with CVD risk score ≥10%; a relative increment of 28% (95% confidence interval: 14–44%, *P* < 0.001).

The adjusted relative odds of elevated CVD risk for opportunistic checks compared with invited were 1.70 (95% confidence interval: 1.45–1.99, *P* < 0.001) (Table 1). Higher proportions with increased CVD risk were consistently observed across sub-groups of gender, age and ethnicity (Table 1). In the most deprived fifth, 106/695 (15.3%) of invited checks and 230/1 028 (22.4%) of opportunistic checks were associated with elevated CVD risk (adjusted odds ratio: 1.94, 1.37–2.74, *P* < 0.001). In the third quintile of deprivation (the highest for which estimation was feasible), similar proportions of invited and opportunistic checks were associated with elevated CVD risk (odds ratio: 1.10, 0.80–1.51, *P* = 0.572). These results show that respondents at opportunistic checks tend to be individuals with higher CVD risk and greater levels of deprivation.

**Table 1:** Proportion with CVD risk score greater than 10% by source of health check. Figures are frequencies except where indicated

Characteristic		Invited health checks			Opportunistic health checks			Relative odds of 10% CVD risk if check is opportunistic (95% confidence interval)	*P* value
		*n*	*N*	**%**	*n*	*N*	**%**		
All		382	2246	(17.0)	692	3113	(22.2)	1.70 (1.45 to 1.99)	<0.001
Gender	Female	106	1210	(8.8)	216	1671	(12.9)	1.85 (1.36 to 2.53)	<0.001
	Male	276	1036	(26.6)	476	1442	(33.0)	1.63 (1.33 to 2.00)	<0.001
Age-group	40–59	206	1880	(11.0)	448	2703	(16.6)	1.66 (1.37 to 2.00)	<0.001
	60–74	176	366	(48.1)	244	410	(59.5)	1.85 (1.33 to 2.59)	<0.001
Ethnicity	White	96	533	(18.0)	179	803	(22.2)	1.49 (1.17 to 1.89)	0.001
	Black	70	551	(12.7)	150	875	(17)	1.74 (1.37 to 2.21)	<0.001
	Asian	25	143	(17.5)	47	217	(21.7)	1.66 (1.03 to 2.69)	0.037
	Mixed	146	789	(18.5)	254	953	(26.7)	1.92 (1.53 to 2.42)	<0.001
	Other	13	59	(22.0)	13	70	(18.6)	1.18 (0.42 to 3.30)	0.752
	Missing	32	171	(18.7)	49	195	(25.1)	1.62 (1.15 to 2.28)	0.005
IMD quintile	Most deprived	106	695	(15.3)	230	1028	(22.4)	1.94 (1.37 to 2.74)	<0.001
	4	202	1197	(16.9)	365	1646	(22.2)	1.68 (1.39 to 2.04)	<0.001
	3	48	235	(20.4)	60	290	(20.7)	1.10 (0.80 to 1.51)	0.572
	2	5	15	(33.3)	0	2	(0)	–	
	Missing	21	104	(20.2)	37	147	(25.2)	–	

Odds ratios were adjusted for each of the variables shown.

Supplementary Table 1 presents the proportion of participants that were overweight or obese by source of check. Overall, 55.7% of participants receiving invited checks and 58.8% of participants receiving opportunistic checks were identified as being overweight or obese (odds ratio: 1.15, 1.04–1.28, *P* = 0.008).

### Sensitivity analyses

As a sensitivity analysis, we omitted all 1 363 ‘opportunistic’ health checks completed within 6 months of the practice joining the study, as these might have been invited in an early period. The adjusted odds ratio for ≥10% CVD risk associated with opportunistic checks was then 1.40 (1.14–1.73, *P* = 0.002), based on 3996 observations. We evaluated the effect of omitting 376 health checks in invited participants that were completed more than 6 months after the invitation, as these might have been opportunistic checks. The adjusted odds ratio was then 1.65 (1.40–1.94, *P* < 0.001), based on 4983 observations. We conclude that the reported association is robust to varying the case definition for an opportunistic check.

We evaluated whether lower ascertainment of risk scores for opportunistic health checks might have influenced the findings. We compared nine practices where CVD risk scores were obtained for more than 85% (median: 91%, range: 86–95%) with nine practices with CVD risk scores obtained for fewer than 85% (median: 76%, range: 33–84%) of opportunistic health checks. Relative increments were similar at 27% (95% confidence interval: 9–49%, *P* = 0.002) and 29% (9–54%, *P* = 0.003) respectively.

## Discussion

### What this study shows

Opportunistic health checks, conducted in patients who have not received standard invitations through the population-based call–recall system, contribute more than half of all health checks completed in the study area. Once opportunistic health checks are accounted for, the true response rate to the standard invitation letter is low. Opportunistic checks represent a higher proportion of all health checks performed in younger adults and in more deprived areas, appearing to compensate for low uptake in these groups. Participants taking up opportunistic checks are at higher risk of cardiovascular disease, suggesting that general practices may be successfully targeting groups that are at greater risk for the offer of an opportunistic health check.

### Comparison with other studies

Previous studies have reported on the problem of low uptake of health checks.^5,13^

Our previous qualitative research,^14^ found that people may experience significant difficulties in gaining access to health checks. For people in work, or with caring responsibilities, it may be difficult or costly to free-up time to have a health check; places where checks are conducted may not be conveniently located in relation to daily activities, and it may be difficult to obtain appointments.^14^ Studies of general practice confirm that there is variability in the organization and delivery of health checks.^15,16^ In the presence of several personal and organizational barriers to obtaining a health check, it may be easier for patients to take up the offer of a check when they are already attending the practice for another reason. Practices may also be able to offer checks at a time when they are able to deliver these.^16^ An opportunistic approach to health checks may offer advantages to patients and providers. While our report refers to general practice health checks, Dachsel and Lee^17^ reported on a pilot study of health checks in a retail environment (supermarket) and noted a high proportion at elevated risk among respondents. Adopting strategies that aim to include higher risk participants is consistent with the recent recommendation of the Expert Scientific and Clinical Advisory Panel for NHS Health Checks that ‘targeting the programme at high-risk people is cost-effective.’^18^

### Strengths and limitations

The study comprised a large sample of general practices across two London boroughs, with data collected and analysed for more than 5000 health checks. Estimated effects were precise and robust in several sensitivity analyses that varied case definitions and tested the potential for bias from missing data. We acknowledge that the study was conducted in a single area. It is possible that associations might differ in other areas. Nevertheless, the results draw attention to the potential of using opportunistic checks to reduce uptake inequalities even if this is not presently performed in other areas. In this study, the majority of the population lived in areas classified in the lowest two quintiles of deprivation for England. The study necessarily had limited scope to explore socioeconomic graduations but we can be confident that our results hold for a generally deprived area. We classified health checks as being recorded ‘opportunistically’ if they were not within 6 months of an invitation letter but this might lead to misclassification. Some patients might respond to an opportunistic request to have a check in a period soon after an invitation, others might take longer than 6 months after an invitation to complete a health check. This misclassification is likely to diminish the magnitude of estimated associations. We also note that misclassification might be more important in the first 6 months of the study because participants might have been responding to invitations in an earlier period. After excluding checks completed in the first 6 months, the adjusted odds ratio remained highly significant through slightly diminished in magnitude. We also obtained data from the district-wide health check management information system, which confirmed that ~50% of all checks were ‘non-invited’, as reported elsewhere.^6^ We noted that more opportunistic health checks were recorded without values being entered for cardiovascular risk scores. This suggests that primary care staff may be less likely to record a CVD risk score when they complete an opportunistic health check. It is possible that scores are less likely to be recorded if they are not elevated and this might be an important form of bias. However, we found that estimated associations were similar at practices with either lower or higher proportions of missing CVD risk scores values. This suggests that reported associations are unlikely to be explained by differential under-ascertainment of ‘normal’ CVD risk scores.

### Conclusions

This study showed that health checks performed opportunistically are reaching participants at higher risk of cardiovascular disease compared to those performed in response to a standard invitation letter. This approach has the potential to reduce inequalities in uptake and outcomes from health checks. Guidelines and policies on cardiovascular health checks should consider using opportunistic checks alongside invitations as an approach to targeting more vulnerable groups to complete a health check. Further research is required to ensure that these groups are also taking up interventions and gaining health benefit from reduced cardiovascular risks.

## Supplementary Material

Supplementary Data

## References

[1] Department of Health. Putting Prevention First NHS health check: vascular risk assessment Best Practice Guidance. London: Department of Health, 2009.

[2] Krogsbøll LT, Jørgensen KJ, Grønhøj Larsen C et al General health checks in adults for reducing morbidity and mortality from disease. Cochrane Database Syst Rev 2012;10:CD009009. 23076952 10.1002/14651858.CD009009.pub2

[3] Imperial Cancer Research Fund Oxcheck Study Group The effectiveness of health checks conducted by nurses in primary care: final results from the OXCHECK study. Br Med J 1995;310:1099–104. 7742676 PMC2549499

[4] Capewell S, McCartney M, Holland W Invited debate: NHS Health Checks—a naked emperor? J Public Health 2015;37:187–92. 10.1093/pubmed/fdv063 26022810

[5] Artac M, Dalton ARH, Majeed A et al Uptake of the NHS health check programme in an urban setting. Fam Pract 2013;30:426–35. 23377607 10.1093/fampra/cmt002 PMC3722503

[6] Forster AS, Burgess C, McDermott L et al Enhanced invitation methods to increase uptake of NHS health checks: study protocol for a randomized controlled trial. Trials 2014;15:342. 25174568 10.1186/1745-6215-15-342 PMC4156615

[7] McDermott L, Wright AJ, Cornelius V et al Enhanced invitation methods and uptake of health checks in primary care: randomised controlled trial and cohort study using electronic health records. Health Technol Assess 2016;20(84):1–92. 10.3310/hta20840 PMC5124783 27846927

[8] National Statistics *Neighbourhood Statistics* London: Office for National Statistics; 2015. http://www.neighbourhood.statistics.gov.uk/dissemination/.

[9] Department for Communities and Local Government English Indices of Deprivation 2010. London: Department for Communities and Local Government, 2011 https://www.gov.uk/government/statistics/english-indices-of-deprivation-2010.

[10] Joint British Societies Joint British Societies’ consensus recommendations for the prevention of cardiovascular disease (JBS3). Heart 2014;100(Suppl. 2):ii1–ii67. 24667225 10.1136/heartjnl-2014-305693

[11] Hippisley-Cox J, Coupland C, Robson J et al Derivation, validation, and evaluation of a new QRISK model to estimate lifetime risk of cardiovascular disease: cohort study using QResearch database. Br Med J 2010;341:c6624. 21148212 10.1136/bmj.c6624 PMC2999889

[12] Garg N, Muduli SK, Kapoor A et al Comparison of different cardiovascular risk score calculators for cardiovascular risk prediction and guideline recommended statin uses. Indian Heart J. In press. 10.1016/j.ihj.2017.01.015 PMC5560874 28822511

[13] Cochrane T, Gidlow CJ, Kumar J et al Cross-sectional review of the response and treatment uptake from the NHS Health Checks programme in Stoke on Trent. J Public Health 2013;35:92–8. 10.1093/pubmed/fds088 PMC3580053 23104892

[14] Burgess C, Wright AJ, Forster AS et al Influences on individuals’ decisions to take up the offer of a health check: a qualitative study. Health Expect 2015;18:2437–48. 24889817 10.1111/hex.12212 PMC5810718

[15] Nicholas JM, Burgess C, Dodhia H et al Variations in the organization and delivery of the ‘NHS health check’ in primary care. J Public Health 2013;35:85–91. 10.1093/pubmed/fds062 22829660

[16] Shaw RL, Lowe H, Holland C et al GPs’ perspectives on managing the NHS Health Check in primary care: a qualitative evaluation of implementation in one area of England. BMJ Open 2016;6(7):e010951. 10.1136/bmjopen-2015-010951. PMC4947777 27388356

[17] Dachsel M, Lee E Opportunistic health checks in a retail environment. London J Prim Care 2011;4:5–10. 10.1080/17571472.2011.11493321 PMC3960676 25949641

[18] Expert Scientific and Clinical Advisory Panel Emerging Evidence on the NHS Health Check: Findings and Recommendations. London: Public Health England, 2017.

