# Supplementary material for: Cardiovascular risk at health checks performed opportunistically or following an invitation letter. Cohort study
Source: J Public Health (Oxf). 2017 Jun 17;40(2):e151–6. doi: 10.1093/pubmed/fdx068 (PMC6053837; doi:10.1093/pubmed/fdx068)
Supplement: Supplementary Data [file jph_16_0547_supplementarytable1.docx]

**Supplementary Table 1: Proportion identified as overweight or obese by source of health check.**

| **Characteristic** |  | **Invited health checks** | | | **Opportunistic health checks** | | | **Odds ratio**  **(95% confidence interval)** | **P value** |
| --- | --- | --- | --- | --- | --- | --- | --- | --- | --- |
|  |  | n | **N** | **%** | n | **N** | **%** |  |  |
|  |  |  |  |  |  |  |  |  |  |
| **All** |  | 1,252 | 2,246 | (55.7) | 1,831 | 3,113 | (58.8) | 1.15 (1.04 to 1.28) | 0.008 |
|  |  |  |  |  |  |  |  |  |  |
| **Gender** | Female | 639 | 1,210 | (52.8) | 969 | 1,671 | (58.0) | 1.25 (1.07 to 1.47) | 0.005 |
|  | Male | 613 | 1,036 | (59.2) | 862 | 1,442 | (59.8) | 1.05 (0.90 to 1.24) | 0.521 |
|  |  |  |  |  |  |  |  |  |  |
| **Age-group** | 40-59 | 1,057 | 1,880 | (56.2) | 1,625 | 2,703 | (60.1) | 1.17 (1.09 to 1.27) | <0.001 |
|  | 60-74 | 195 | 366 | (53.3) | 206 | 410 | (50.2) | 1.04 (0.67 to 1.62) | 0.861 |
|  |  |  |  |  |  |  |  |  |  |
| **Ethnicity** | White | 268 | 533 | (50.3) | 439 | 803 | (54.7) | 1.30 (1.08 to 1.57) | 0.007 |
|  | Black | 387 | 551 | (70.2) | 626 | 875 | (71.5) | 1.05 (0.82 t0 1.34) | 0.687 |
|  | Asian | 54 | 143 | (37.8) | 104 | 217 | (47.9) | 1.40 (0.81 to 2.40) | 0.228 |
|  | Mixed | 406 | 789 | (51.5) | 501 | 953 | (52.6) | 1.11 (0.92 to 1.33) | 0.299 |
|  | Other | 41 | 59 | (69.5) | 42 | 70 | (60.0) | 0.48 (0.24 to 0.97) | 0.040 |
|  | Missing | 96 | 171 | (56.1) | 119 | 195 | (61.0) | 1.28 (0.76 to 2.14) | 0.349 |
|  |  |  |  |  |  |  |  |  |  |
| **IMD quintile** | Most deprived | 421 | 695 | (60.6) | 645 | 1,028 | (62.7) | 1.12 (0.90 to 1.40) | 0.291 |
|  | 4 | 650 | 1,197 | (54.3) | 946 | 1,646 | (57.5) | 1.19 (1.05 to 1.34) | 0.005 |
|  | 3 | 121 | 235 | (51.5) | 162 | 290 | (55.9) | 1.25 (0.78 to 2.01) | 0.363 |
|  | 2 | 6 | 15 | (40) | 0 | 2 | (0) | - |  |
|  | Missing | 54 | 104 | (51.9) | 78 | 147 | (53.1) | - |  |
|  |  |  |  |  |  |  |  |  |  |

189 participants had no BMI values recorded
